# Sequestration of Ribosome during Protein Aggregate Formation: Contribution of ribosomal RNA

**DOI:** 10.1038/srep42017

**Published:** 2017-02-07

**Authors:** Bani K. Pathak, Surojit Mondal, Senjuti Banerjee, Amar Nath Ghosh, Chandana Barat

**Affiliations:** 1 Structural Biology & Bio-Informatics Division, Indian Institute of Chemical Biology (Council of Scientific & Industrial Research), 4, Raja S.C. Mullick Road, Kolkata, 700 032, India; 2 Krish Biotech Research Pvt. Ltd. T-1, QK-17 (Part) WBIIDC, Kalyani, Phase III, Nadia West Bengal 741 235, India; 3 Department of Biotechnology, St. Xavier’s College, Park Street, Kolkata-700016, West Bengal, India; 4 National Institute of Cholera and Enteric Diseases P-33, C.I.T. Road, Scheme XM, Beleghata, India

## Abstract

An understanding of the mechanisms underlying protein aggregation and cytotoxicity of the protein aggregates is crucial in the prevention of several diseases in humans. Ribosome, the cellular protein synthesis machine is capable of acting as a protein folding modulator. The peptidyltransferase center residing in the domain V of large ribosomal subunit 23S rRNA is the centre for the protein folding ability of the ribosome and is also the cellular target of several antiprion compounds. Our *in vitro* studies unexpectedly reveal that the partial unfolding or aggregation of lysozyme under reducing conditions in presence of the ribosome can induce aggregation of ribosomal components. Electrostatic interactions complemented by specific rRNA-protein interaction drive the ribosome-protein aggregation process. Under similar conditions the rRNA, especially the large subunit rRNA and *in vitro* transcribed RNA corresponding to domain V of 23S rRNA (bDV RNA) stimulates lysozyme aggregation leading to RNA-protein aggregate formation. Protein aggregation during the refolding of non-disulfide containing protein BCAII at high concentrations also induces ribosome aggregation. BCAII aggregation was also stimulated in presence of the large subunit rRNA. Our observations imply that the specific sequestration of the translation machine by aggregating proteins might contribute to their cytotoxicity.

Protein aggregation has been recognized as a hallmark of numerous disease conditions in humans such as Alzheimer, Parkinson, Type II Diabetes and prion diseases^1^. Understanding the mechanisms underlying protein misfolding and aggregation has thereby become a central issue in biology and medicine^2^. Several factors such as the molecular chaperones and protein degradation machinery act as cellular protection mechanisms against the accumulation of protein aggregates. The identification of these factors and the factors that are responsible for toxicity of the protein aggregates are crucial in the prevention of disease in normally functioning living organisms.

Ribosome, the cellular protein synthesis machine is also the platform for folding of newly synthesized polypeptide chains^3^,^4^. The electrostatics of the ribosome surface also influences the dynamics of the nascent proteins^5^. In addition, the ability of the ribosome to itself act as a protein folding modulator has been demonstrated with ribosomes derived from varied sources (eubacteria like *E. coli* and *B. subtilis*, archaebacteria; eukaryotes like yeast, rat liver, wheat germ and bovine mitochondria) and with proteins belonging to wide range of structural classes (ref. 6 and the references therein). The protein folding activity resides at the peptidyl transferase center (PTC) in the domain V of large ribosomal subunit 23S rRNA in prokaryotic ribosomes^7^,^8^. The *in vitro* transcribed RNA corresponding to the domain (bDV RNA) also exhibits this activity. Studies on the mechanisms of bDV RNA protein folding activity showed that it is a two-step process involving its two sub-domains RNA1 and RNA2. The initial binding of the unfolded proteins takes place with the RNA1 sub-domain and the RNA2 region of this domain is responsible for releasing the bound protein which subsequently folds into its native structure^9^. The domain V of large subunit rRNA of bovine mitochondrial ribosome (mDV RNA) has a truncated RNA2 region and therefore shows a delay in releasing the bound protein^10^. It has been proposed that a set of five conserved nucleotides in the RNA1 sub-domain interacts identically with the nascent and chemically unfolded proteins *in vivo* and *in vitro* leading to nucleation of folding of the nascent polypeptide chain synthesized by the ribosome^11^,^12^. Interestingly, several studies have identified the ribosomal RNA as the cellular target of two antiprion compounds, 6-Aminophenanthridine (6AP) and Guanabenz acetate (GA)^13^,^14^,^15^,^16^,^17^. The compound 6AP has a well-documented antiprion activity but does not interact directly with the prion protein. Instead it interacts with the identical set of five nucleotides on the domain V of 23S rRNA that are necessary for interaction with protein substrates during the process of ribosome modulated protein folding. Although the mechanism by which 6AP inhibits prion phenotype is not yet understood, it has been suggested that the rRNA might indirectly facilitate prion propagation by either conversion of the prion proteins to the amyloid forms or by breaking long prion fibrils into smaller prion seeds^15^,^16^. A recent study has demonstrated a direct involvement of the protein folding activity associated with the ribosome (PFAR) in yeast prion propagation^18^. This study has also provided insight into involvement of the ribosome in the basal thermotolerance and protein refolding after heat shock in yeast cells. These studies might also suggest the involvement of the protein folding activity of the ribosome in varied aspects of cellular protein folding and aggregation processes. Our initial studies demonstrated that the ribosome and *in vitro* transcribed bDV RNA could bind to and interfere in the aggregation of partially folded BCAII (bovine carbonic anhydrase II) and reduced denatured hen egg white lysozyme (lysozyme)^19^. Our present study was initiated to investigate the effect of the bacterial ribosome or the ribosomal RNA on aggregation of lysozyme under reducing conditions.

Lysozyme is a small globular protein with 4 disulfide linkages. Since its pI is 11.35, it is predominantly positively charged at physiological pH. This protein has been extensively used to understand the mechanism of protein folding, misfolding and amyloid fibril formation^20^. It has also been reported that the lysozyme fibrils are toxic to cell cultures^21^. In addition, hen egg white lysozyme possesses a high degree of sequence and structural homology with human lysozyme, which is associated with systemic amyloidosis in humans^22^. When lysozyme is exposed to high concentration of the reducing agent DTT, disulfide bond breakage and scrambling occurs leading to the formation of loosely packed amorphous aggregates^23^. Our studies indicate that partial unfolding or amorphous aggregation of lysozyme in the presence of empty non-translating prokaryotic or eukaryotic ribosome could induce aggregation of ribosomal components. The ability to induce ribosome aggregation has also been demonstrated with BCAII, a protein with no disulfide linkages. Subsequent studies implicating a role of the ribosomal RNA in the ribosome-protein aggregation process is also discussed here.

## Results

As stated earlier, when lysozyme is exposed to high concentration of the reducing agent DTT, disulfide bond breakage and scrambling leads to the formation of loosely packed amorphous aggregates^23^. Our initial studies were aimed at determining the effect of the ribosome on lysozyme aggregation under reducing conditions. We therefore followed the aggregation process by using turbidity measurements at 450 nm or agarose and polyacrylamide gel electrophoresis.

### Effect of ribosome on lysozyme aggregation

**Figure 1:**
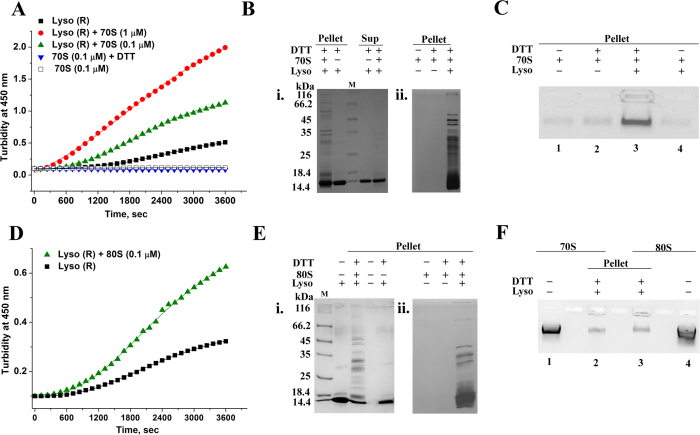
Effect of ribosome on lysozyme aggregation. Aggregation of lysozyme in presence of 70S ribosome was followed by studying time course of change in turbidity and SDS-PAGE or agarose gel electrophoresis. For gel electrophoresis experiments, the samples loaded in each lane are depicted in panels above the gels. Lysozyme in presence of 20 mM DTT is depicted as Lyso (R). (**A**) Time course of change in turbidity when 10 μM lysozyme was incubated with 20 mM DTT in absence (◾) and presence of different concentrations of 70S ribosome: 1 μM 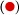, 0.1 μM 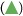. In the control experiment, change in turbidity was measured in presence of 70S (0.1 μM) (◽) and 70S (0.1 μM) + DTT 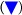. (**B**)i) The aggregate pellet obtained by centrifugation and concentrated supernatant from incubation of 10 μM of lysozyme + 20 mM DTT + 0.1 μM 70S were loaded on a 12% SDS-PAGE for 45 minutes at room temperature (Materials and Methods). ii) Pellets from 70S alone, 70S + DTT and 70S + DTT + lysozyme were run on a 12% SDS PAGE. (**C**) 0.1 μM 70S ribosome was incubated with and without of 10 μM lysozyme in the absence or presence of 20 mM DTT in Buffer A, was centrifuged and the resuspended pellet (in Buffer A containing 1 M urea) loaded on a 1% agarose gel. (**D**) Time course change in turbidity when 10 μM of lysozyme was incubated with substoichiometric amount of yeast 80S ribosome (0.1 μM) in presence of 20 mM DTT: lysozyme + DTT (◾) and lysozyme + DTT + 80S 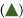. (**E**) 10 μM of lysozyme was incubated with 20 mM DTT in the presence of 0.1 μM 80S ribosome or in the absence of 80S ribosome, centrifuged, pellet resuspended in 40 μl of Buffer A and loaded on the 12% SDS – PAGE. F) 10 μM of lysozyme was incubated with 20 mM DTT in the presence of either 0.1 μM 70S or 0.1 μM 80S ribosome, centrifuged; the pellet resuspended in 25 μl of Buffer A containing 1 M urea and loaded on 1% agarose gel.

Lysozyme (10 μM), was incubated in 20 mM DTT at physiological pH (pH 7.5; Buffer A) in the absence or in the presence of the 70S ribosome (Methods section) and the change in turbidity was followed for a period of 60 min. As shown in Fig. 1, while a moderate increase in turbidity was observed with lysozyme alone, a significant increase was observed (Fig. 1A), even when the ribosome was present at concentrations 10-fold (1 μM) or 100-fold (0.1 μM) lower with respect to lysozyme (10 μM). No increase in turbidity was observed when 0.1 μM of 70S was incubated alone or in presence of DTT.

The constituents of the aggregate then needed to be further analysed. The aggregate formed upon incubation of lysozyme (10 μM) and ribosome (0.1 μM) for 45 min under reducing conditions was separated by centrifugation (Methods section). The aggregate pellet and the supernatant were analysed by denaturing polyacrylamide gel electrophoresis. Unexpectedly, as shown in Fig. 1B(i), a significant fraction of the ribosomal proteins appeared in the aggregate with their concomitant reduction in equivalent amount in the supernatant. Since several ribosomal proteins co-migrate with lysozyme in SDS-PAGE, the intensity of the lysozyme band does not reflect the change in level of aggregation of the protein itself in presence of the ribosome. However, the contribution of the large number of ribosomal proteins per ribosome in the aggregate did explain the increase in turbidity observed even when lysozyme was incubated with sub-stoichiometric concentrations of the ribosome (1:100) (Fig. 1A). Agarose gel electrophoresis of the aggregate pellet resuspended in Buffer A containing 1 M urea, showed that ribosomal RNA was also a component of the aggregate formed. Hence, in our subsequent studies the extent of ribosomes partitioning in the insoluble aggregate has been estimated by the intensity of the rRNA band observed upon agarose gel electrophoresis of the aggregate. As shown in Fig. 1C, no rRNA was present in the aggregate when native lysozyme is incubated with the ribosome in absence of DTT. Also, no ribosomal proteins or rRNA were present in the pellet fraction when 70S ribosome was either incubated alone or in presence of DTT under the conditions stated above (Fig. 1B,C). Hence, the interaction of ribosome with partially unfolded or aggregated lysozyme (due to breaking of disulphide linkage in presence of DTT) is necessary for lysozyme induced ribosome aggregation. The presence of DTT or the conditions used in the experiment did not contribute to the ribosome aggregation observed here. Earlier studies have also demonstrated that the ribosome remains intact and therefore translationally active even in presence of 30 mM DTT^24^.

Our next set of experiments was performed to study the effect of lysozyme on the eukaryotic yeast 80S ribosome under reducing conditions. Incubation of lysozyme (10 μM) with substoichiometric concentration (0.1 μM) of yeast 80S ribosome under reducing conditions also showed enhanced increase in turbidity and presence of ribosomal components in the aggregates by SDS-PAGE analysis (Fig. 1E and lane 3 of Fig. 1F). This study demonstrated that the eukaryotic yeast ribosome is also susceptible to lysozyme induced aggregation under reducing conditions. Control experiments were performed in which the 80S ribosome was similarly incubated in presence and absence of DTT, centrifuged and the pellets were analysed by SDS PAGE. The absence of ribosomal proteins in the pellet confirmed that treatment of the ribosome with DTT alone does not lead to ribosomal aggregate formation.

Next, to determine the extent of ribosome aggregation with respect to the total amount of ribosome present in the experiment, 0.1 μM of the 70S or 80S ribosome was incubated with lysozyme (10 μM) in presence of DTT (Fig. 1F). The rRNA in the aggregate formed in presence of 70S ribosome and 80S ribosome was found to be comparable (Lanes 2 and 3). The intensity of the rRNA band in the aggregate was significantly less as compared to the total rRNA present in the experiment (from 0.1 μM ribosome). This showed that at the concentration of lysozyme used in this study (10 μM), only a fraction of the total ribosomal RNA (Lanes 1 and 4) partitioned into the aggregate formed (Lanes 2 and 3 of Fig. 1F).

### Study of time course of lysozyme-ribosome coaggregation

**Figure 2:**
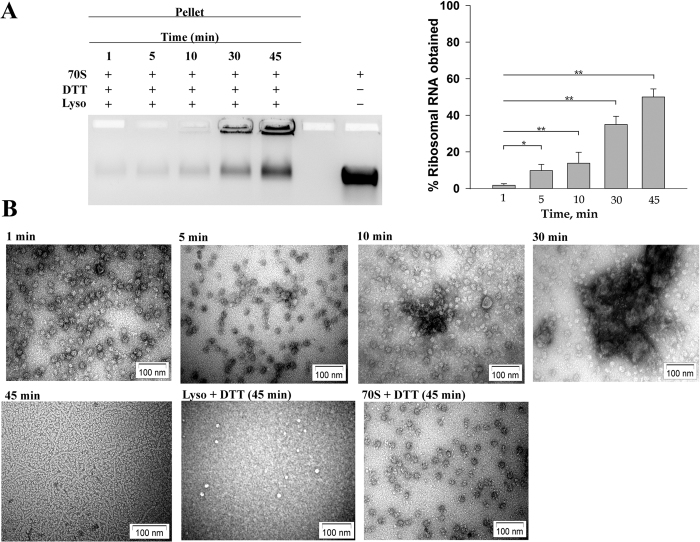
Time course of lysozyme-ribosome aggregation. (**A**) 0.1 μM 70S ribosome was incubated in 20 mM DTT in the presence of 10 μM lysozyme for different time intervals as indicated in the figure, centrifuged and the resuspended pellets were loaded on the 1% agarose gel. 0.1 μM 70S ribosome that was loaded in the last lane represents the total ribosomal RNA present in each experiment. Densitometry analysis of the rRNA bands corresponding to each time points were expressed as percentage of total ribosomal RNA present and depicted as bar graphs. Data are presented as means ± SEM; *P < 0.05 or **P < 0.001 in one –way ANOVA (N = 5). (**B**) Micrographs were prepared from samples withdrawn at different time intervals from the initiation of incubation of 10 μM of lysozyme and 0.1 μM of 70S ribosome in presence of 20 mM DTT: after 1 min, after 5 min, after 10 min, after 30 min and 45 minutes of incubation. The control micrographs represent lysozyme or 70S ribosome that were incubated separately in 20 mM DTT for 45 min.

To follow the time course of the ribosome aggregation, lysozyme (10 μM) was incubated with 70S ribosome (0.1 μM) in presence of DTT in separate reactions. The reaction mix was centrifuged at different time intervals and the rRNA in the aggregate analysed by agarose gel electrophoresis. The intensity of the RNA band in the aggregate would be a measure of the extent of ribosome aggregation induced at the specified time points (Fig. 2A). A semi-quantitative representation of the intensities of the rRNA bands is also shown in the figure as a bar diagram. Although accurate estimation of rRNA in the aggregate was not possible due to the large amount of rRNA being still retained in the lanes of the agarose gel, this figure shows that, the process of appearance of the ribosome in the aggregate fraction is significantly enhanced between the 10 min and 30 min time points after the start of incubation. Electron microscopic study was also performed to follow the time course of lysozyme induced ribosome aggregation. An initial clumping of the ribosome occurs at 1 min, followed by progressive loss of ribosomal integrity from 5 min to 30 min time interval, ultimately resulting in aggregation of the ribosomal components by 45 min after initiation of interaction (Fig. 2B). The electron microscopy of 70S ribosome (0.1 μM) or lysozyme (10 μM) incubated in presence of DTT for 45 min was also performed (Fig. 2B). No significant aggregate formation was observed when lysozyme or the ribosome was incubated alone in presence of DTT.

### Effect of lysozyme concentration on the protein-ribosome aggregation process

**Figure 3:**
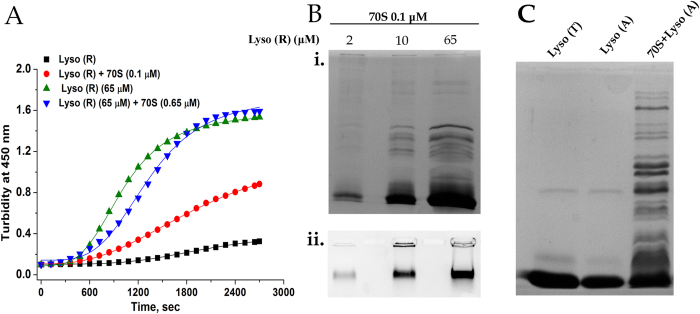
Effect of lysozyme concentration on the protein-ribosome coaggregation process. (**A**) Time course of change in turbidity when lysozyme at two different protein concentrations was incubated with 20 mM DTT in absence and presence of 70S ribosome: 10 μM lysozyme + 20 mM DTT (◾), 65 μM lysozyme + 20 mM DTT 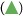, 10 μM lysozyme + 20 mM DTT + 0.1 μM 70S 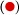, 65 μM lysozyme + 20 mM DTT + 0.65 μM 70S 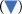. Lysozyme reduced with 20 mM DTT has been abbreviated as Lyso (R). (**B**)i) 2 μM, 10 μM or 65 μM of lysozyme was incubated in 20 mM DTT in the presence of 0.1 μM 70S ribosome for 45 minutes at room temperature, centrifuged and the resuspended pellets (in 40 μl of Buffer **A**) were loaded on a 12% SDS PAGE. Lysozyme reduced with 20 mM DTT has been abbreviated as Lyso (R). ii) 2 μM, 10 μM or 65 μM of lysozyme was incubated in 20 mM DTT in the presence of 0.1 μM 70S ribosome for 45 minutes at room temperature, pelleted down and the resuspended pellets (in 25 μl Buffer A containing 1 M urea) were loaded on a 1% agarose gel. Lanes from left to right for both the gels contain: 2 μM lysozyme + 20 mM DTT + 70S, 10 μM lysozyme + 20 mM DTT + 70S, 65 μM lysozyme + 20 mM DTT + 70S. (**C**) 65 μM of lysozyme was incubated in 20 mM DTT for 30 min, centrifuged and the pellet obtained was incubated in Buffer A with or without 0.65 μM 70S in the presence of DTT for 45 minutes at room temperature. After incubation samples were centrifuged, aggregate resuspended in 40 μl of Buffer A and loaded on a 12% SDS-PAGE. Left to right lane: Lyso (T): 65 μM lysozyme (Total protein), Lyso (A): aggregate of 65 μM of lysozyme incubated with 20 mM DTT for 30 min (pellet), 70S + Lyso (A): Lyso (A) + 20 mM DTT + 0.65 μM 70S (pellet).

It is well established that an increase in protein concentration leads to increased protein aggregation. Hence, further studies were conducted to observe whether the facilitation of protein-protein interaction at higher protein concentration could lead to reduced lysozyme-ribosome interaction and hence reduced ribosome aggregation. As shown in Fig. 3A, a comparable increase in turbidity was observed when lysozyme at a concentration of 65 μM was incubated in 20 mM DTT either in the absence or in the presence of the ribosome (0.65 μM) (Fig. 3A).This initially suggested that the ribosome aggregation was circumvented at high protein concentrations. However, SDS-PAGE analysis still revealed the presence of ribosomal components in the aggregate formed when 2 μM, 10 μM or 65 μM of lysozyme (with DTT) was incubated with 0.1 μM of 70S ribosome. This observation suggested that turbidity measurements at 450 nm were unable to register the lysozyme induced ribosome aggregation at higher protein concentrations (Fig. 3Bi). Also, the relative intensity of the rRNA in the aggregate obtained upon incubation of 2 μM, 10 μM or 65 μM lysozyme with equivalent amount of ribosome (0.1 μM), showed increasing rRNA aggregation with increasing protein concentrations (Fig. 3Bii). This is possible either if ribosome-protein interaction leading to ribosomal aggregation supersedes increased protein-protein interaction or if the aggregate formed is also capable of inducing ribosome trapping and hence its aggregation.

To test whether a preformed protein aggregate is also capable of triggering the ribosome aggregation process, 65 μM lysozyme was incubated in 20 mM DTT for 30 min. Lysozyme undergoes almost complete aggregation under these conditions. The lysozyme aggregates obtained by centrifugation was incubated with 0.65 μM ribosome for 45 minutes. SDS PAGE analysis shows that the preformed lysozyme aggregates also had the ability to stimulate ribosome aggregation (Fig. 3C). The loose and flexible structure of lysozyme aggregates formed under reducing conditions^23^ could have enabled protein-ribosome interaction to occur and thereby induced ribosome aggregation. This ability of lysozyme aggregates might contribute significantly to the increased ribosome aggregation observed at high protein concentration (Fig. 3B) or on a longer time scale at low protein concentration as shown in Fig. 2A. A recent study has also demonstrated that the addition of pathological tau oligomers in an *in vitro* translation assay leads to significant reduction in translation of the GFP reporter gene and this observation has been attributed to a dysfunctional consequence of tau–ribosome association in Alzheimer’s patients^25^.

### Effect of heparin and tRNA in lysozyme-ribosome aggregation

**Figure 4:**
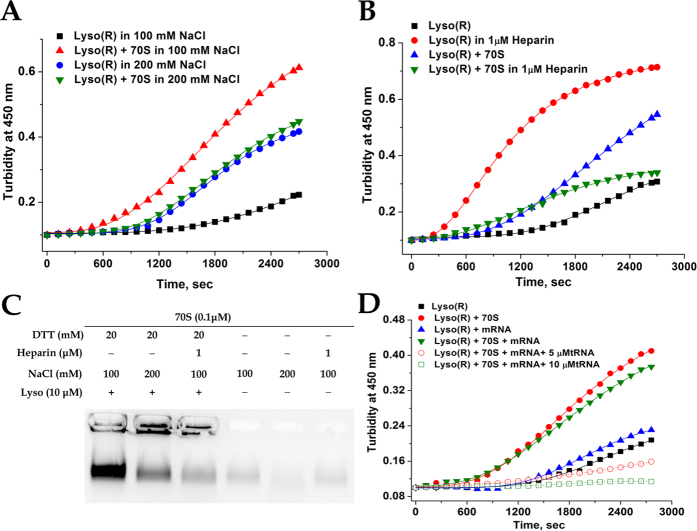
Effect of Salt, heparin and tRNA on lysozyme-ribosome aggregation. Aggregation of lysozyme in presence of 70S ribosome and NaCl, heparin, tRNA and mRNA was followed by studying time course change in turbidity and agarose gel electrophoresis. For gel electrophoresis experiments, the samples loaded in each lane are depicted in panels above the gels. Lysozyme in presence of 20 mM DTT is depicted as Lyso (R). (**A**) Time course change in turbidity when 10 μM of lysozyme was incubated with 70S ribosome (0.1 μM) in presence of 20 mM DTT at different NaCl concentrations: lysozyme + 20 mM DTT in 100 mM NaCl (◾), lysozyme + 20 mM DTT in 200 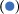, lysozyme + 20 mM DTT + 70S (0.1 μM) in 100 mM NaCl 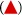, lysozyme + 20 mM DTT + 70S (0.1 μM) in 200 mM NaCl 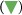. (**B**) Time course change in turbidity when 10 μM lysozyme was incubated with and without 0.1 μM 70S ribosome in 20 mM DTT in presence of 1 μM heparin; lysozyme + 20 mM DTT (◾), lysozyme + 20 mM DTT + heparin 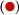, Lysozyme + 20 mM DTT + 70S 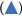, Lysozyme + 20 mM DTT + 70S + heparin 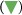. (**C**) 0.1 μM 70S ribosome was incubated with and without 10 μM lysozyme in presence and absence of 20 mM DTT in Buffer A (containing 100 mM or 200 mM NaCl) or Buffer A (containing 1 μM heparin) and centrifuged. The pellets were resuspended in Buffer A containing 1 M urea and run on a 1% agarose gel. (**D**) Time course change in turbidity when 10 μM lysozyme was incubated with 20 mM DTT and 0.1 μM 70S ribosome in absence or in presence of mRNA (0.2 μM) or tRNA: The aggregation process was monitored for lysozyme + 20 mM DTT (◾), Lysozyme + 20 mM DTT + 70S ribosome 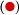, Lysozyme + 20 mM DTT + mRNA 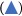, Lysozyme + 20 mM DTT + 70S + mRNA 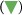, Lysozyme + 20 mM DTT + 70S + mRNA + 5 μM tRNA 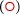, Lysozyme + 20 mM DTT + 70S + mRNA + 10 μM tRNA 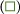.

Lysozyme is predominantly positively charged at the pH used in our study and the ribosome surface has a highly negative electrostatic potential arising primarily from the phosphodiester backbone of the RNA^5^,^26^. Hence, the contribution of electrostatic interaction in lysozyme-ribosome aggregation was studied at high salt concentration and in the presence of a biologically relevant polyanion, heparin. Earlier reports show that heparin causes instant aggregation of denatured and native lysozyme *in vitro*^27^. Turbidity measurements (Fig. 4A) showed that although in the presence of 200 mM NaCl, there was a stimulation of lysozyme aggregation, the lysozyme-ribosome aggregation is significantly suppressed to that in presence of 100 mM NaCl (Buffer A). Similarly, while lysozyme aggregation is itself stimulated in the presence of heparin (1 μM), the lysozyme-ribosome aggregation is suppressed (Fig. 4B). Suppression of ribosome aggregation in presence of high salt concentration or heparin was substantiated by decreased intensity of the ribosomal RNA in agarose gel electrophoresis of the aggregate (Fig. 4C: Lanes 1, 2 and 3). Hence, despite of the fact that presence of heparin and higher salt concentration leads to increased lysozyme aggregation, the shielding of the electrostatic attraction between positively charged lysozyme and the anionic surface of the ribosome could underlie the observed suppression of ribosomal aggregation. This study therefore highlights the importance of electrostatic interactions in the aggregation process. Control experiments were performed in which the 70S ribosome (0.1 μM) was incubated alone with 100 mM NaCl, 200 mM NaCl or 1 μM heparin, centrifuged and the pellets were analysed by agarose gel electrophoresis. The absence of rRNA in the gel confirmed that the aggregation of ribosome is not an effect of either NaCl or heparin used in the experiment but occurs only in presence of lysozyme under reducing conditions (Fig. 4C).

Our next objective was to investigate the effect of ribosome associated polyanions like mRNAs and tRNAs on lysozyme induced ribosome aggregation. The model mRNA used in this experiment contains a Shine Dalgarno sequence that would position an AUG codon at the P-site of the ribosome. In our earlier studies, this mRNA was used to target the binding of deacylated Met-tRNA to the P-site under appropriate conditions^28^. The mRNA concentration used was 0.2 μM and as shown in Fig. 4D, that despite of being polyanionic, the mRNA itself had no significant effect on either lysozyme or lysozyme-ribosome aggregation. Incubation of lysozyme under reducing condition with the mRNA programmed ribosome in presence of deacylated Met- tRNA, led to complete suppression of turbidity increase at both the concentrations of the tRNA used in the experiment (Fig. 4D). Control experiments confirmed that no increase in turbidity was observed when lysozyme was incubated in presence of tRNA or the model mRNA for the duration of the experiments (Figure S2A). However incubation of lysozyme with non-programmed 70S ribosome and tRNA alone also led to suppression of increase in turbidity implying that the positioning of the tRNA at the P-site might not play a significant role in the tRNA mediated aggregation suppression (Figure S2A). This study raises the possibility that for protein-ribosome aggregation to occur, the large electrostatic attractive component between positively charged partially folded lysozyme and the negatively charged ribosomal surface must also be complemented by additional non electrostatic interaction of specific nature. This observation implies that a tRNA bound translating ribosome might be able to evade the aggregation process, although further experiments need to be performed to confirm the same.

### Ribosomal RNA and the Domain V of 23S rRNA stimulate lysozyme aggregation

**Figure 5:**
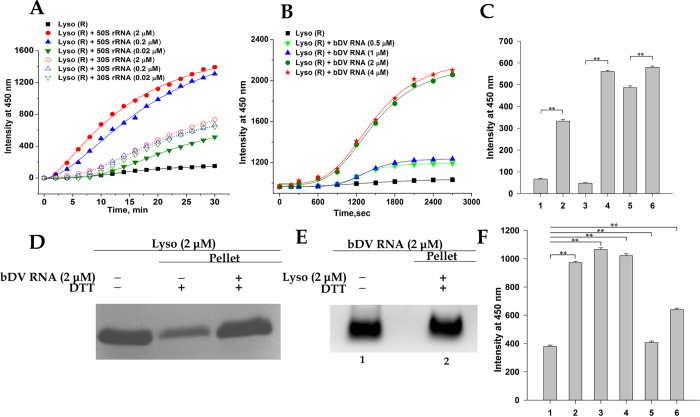
Domain V of 23S rRNA stimulates lysozyme aggregation. Aggregation of lysozyme in presence of rRNA was followed by measuring time course change in light scattering and agarose gel electrophoresis. (Lysozyme + 20 mM DTT) has been abbreviated as Lyso (R). (**A**) Time course of change in light scattering intensity of 2 μM lysozyme in 20 mM DTT in presence of rRNA derived from 50S and 30S ribosomal subunits. The aggregation process of lysozyme (◾) and lysozyme + 50S rRNA [0.02 μM 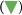, 0.2 μM 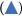, 2 μM 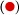]; lysozyme + 30S rRNA [0.02 μM 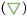, 0.2 μM 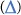, 2 μM 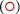] were monitored. (**B**) Comparison of aggregation stimulating activity of bDV RNA on lysozyme (2 μM): The aggregation process of lysozyme (2 μM) (◾); lysozyme + bDV RNA [0.5 μM 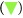, 1 μM 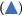, 2 μM 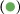 and 4 μM 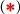]. (**C**) The increase in light scattering intensity in 45 min when lysozyme (in 20 mM DTT) at three different protein concentrations were incubated with stoichiometric concentrations of bDV RNA: 2 μM lysozyme in the absence (1) and presence of bDV RNA (2); 4 μM lysozyme in the absence (3) and presence of bDV RNA (4); 6 μM lysozyme in the absence (5) and presence of bDV RNA (6). Data are presented as means ± SEM. **(p < 0.001, one-way ANOVA, N = 5) shows statistically significant differences in scattering intensity between presence and absence of bDV RNA. (**D** and **E**) 2 μM of lysozyme was incubated in 20 mM DTT with and without 2 μM bDV RNA, centrifuged and the pellet resuspended in Buffer A and loaded on E) 12% SDS–PAGE and F) 1% agarose gel. Samples loaded in each lane are depicted in panels above the gels. (**F**) The increase in light scattering intensity in 45 min when lysozyme (2 μM) in 20 mM DTT were incubated in absence (1) or in presence of equimolar bDV RNA (2), mDV RNA (3), RNA1 (4), RNA2 (5), heparin (6). Statistical significance is shown by **(p < 0.001, one-way ANOVA, N = 5) compared to control (1).

Since the rRNA plays a major role in determining the anionic surface of the ribosome^5^,^26^ and the majority of the interacting sites of tRNA positioned at the P-site also lie on the 23S rRNA^29^, further studies were performed to determine the effect of rRNA extracted from the ribosome or its subunits on lysozyme aggregation under reducing conditions. When lysozyme (2 μM) was incubated with rRNA extracted from both the subunits of prokaryotic ribosome at different stoichiometric concentrations, the aggregation of lysozyme was stimulated to different extents as shown in Fig. 5A. The large ribosomal subunit rRNA (23S rRNA + 5S rRNA) is more effective and shows a dose dependent stimulation of lysozyme aggregation compared to the 16S rRNA extracted from the 30S ribosome. It should however be noted that a stimulation of aggregation is also observed in presence of 30S subunit derived 16S rRNA. The electrostatic nature of the ribosome-lysozyme interaction as stated above might explain why lysozyme, that carries a net positive charge under our experimental conditions, could also interact with the negatively charged 16S rRNA thereby facilitating aggregation via formation of RNA-protein aggregate.

The peptidyltransferase center that resides in the domainV of the 23S rRNA is both the major site of interaction of the tRNA with the 50S subunit^29^ and is also the only domain with a demonstrated ability to interact with unfolded proteins (which form the basis of protein folding activity associated with the ribosome as stated above)^6^,^7^,^8^,^9^,^10^,^11^,^12^. Since lysozyme-ribosome aggregation was suppressed upon tRNA binding and the large subunit rRNA efficiently stimulated lysozyme aggregation, our next objective was to study the effect of *in vitro* synthesized bDV RNA on the aggregation of lysozyme under reducing conditions. Light scattering studies showed that when lysozyme (2 μM) was incubated in 20 mM DTT, stoichiometric concentrations of the bDV RNA could stimulate protein aggregation (Fig. 5B). The aggregation stimulating effect of stoichiometric concentrations of bDV RNA was also observed in experiments conducted in the presence higher lysozyme concentration (4 μM and 6 μM; Fig. 5C). Hence, the presence of bDV RNA is capable of assisting in protein aggregation even under conditions in which the process is facilitated due to increased protein concentrations (Fig. 5C).

The aggregate obtained upon centrifugation (stated above) of lysozyme (2 μM) in presence of bDV RNA (2 μM) was analysed by SDS-PAGE and agarose gel electrophoresis. As shown in Fig. 5D,E, the RNA-protein aggregate formed constituted of almost all the protein and the RNA present in the experiment, thereby providing further evidence of RNA induced aggregation stimulation.

To check whether any entity other than the rRNA could also stimulate lysozyme aggregation the following experiment was performed. Lysozyme aggregation at 10 μM concentration was allowed to proceed in 20 mM DTT in the presence of increasing concentration of native BCAII. The aggregation mix was centrifuged after 45 minutes (Methods) and the supernatants and the pellets were analysed separately on an SDS PAGE. As shown in Figure S1 the amount of aggregated lysozyme remains unchanged in presence of increasing concentrations of BCAII. It should also be noted that the BCAII protein unlike the rRNA did not co-aggregate with the lysozyme and remained in the supernatant. Hence lysozyme aggregation stimulation observed in presence of rRNA was not triggered or stimulated by molecular crowding due to the presence of additional entities.

Earlier studies have demonstrated that the RNA1 sub-domain of bDV RNA is involved in the initial interaction with unfolded polypeptide chain and the RNA2 sub-domain enables the subsequent release of the protein^6^. Truncations in the RNA2 sub-domain of mDV RNA delays the release of the bound protein compared to bDV RNA^10^.

The bar diagram in Fig. 5F shows the increase in light scattering at 450 nm observed upon incubation of lysozyme with *in vitro* transcribed bDV RNA, mDV RNA, RNA1, RNA2 and heparin for 45 min. Similar experiment was also performed in presence of a 165 nucleotides long mRNA corresponding to the rmf (Ribosome Modulation Factor) gene (Materials and methods). The comparable aggregation stimulating effect of mDV RNA, bDV RNA and the RNA1 sub-domain alone indicated that this effect depended only on binding of the RNA to the protein and not on its subsequent release. Also as shown in Fig. 5D and S2B, presence of either heparin or the rmf mRNA had only a marginal effect on lysozyme aggregation. Control experiments confirmed that the rRNA derived from the 50S subunit, 30S subunit and the *in vitro* transcribed bDV RNA or its derivatives themselves did not scatter light in presence of 20 mM DTT for the duration of the experiments (Figure S2C).

**Figure 6:**
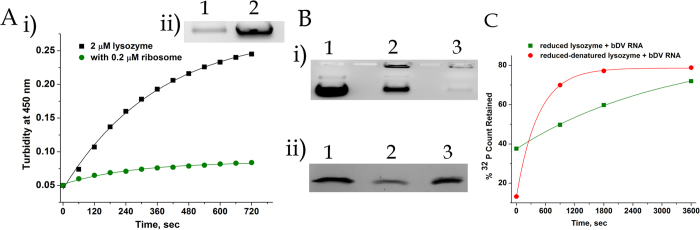
Aggregation and RNA-protein interaction studies with reduced and reduced-denatured lysozyme. (**A**) (i) Time course of change in turbidity of reduced and denatured lysozyme (2 μM) without ribosome (◾) and with 0.2 μM of 70S ribosome 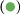. (ii) 2 μM of reduced denatured lysozyme was incubated with 0.2 μM 70S ribosome in Buffer A for 45 minutes, pelleted down and the pellet was resuspended in 25 μl of the same buffer containing 1 M urea and run on a 1% agarose gel. Lane 1, pellet of reduced denatured lysozyme in presence of 70S; lane 2, total 70S rRNA (0.2 μM). (**B**)(i) Reduced and denatured lysozyme (2 μM) were incubated with stoichiometric amounts of bDV RNA (2 μM) for 45 minutes, centrifuged and the pellet was resuspended in 25 μl of Buffer A containing 1 M urea. The total RNA and the resuspended pellets were then loaded on a 1% agarose gel. Lane 1, total bDV RNA; lane 2, lysozyme + 20 mM DTT + bDV RNA (pellet); lane 3, reduced and denatured lysozyme + bDV RNA (pellet). (ii) Reduced and denatured lysozyme (2 μM) or lysozyme (2 μM) in presence of 20 mM DTT were incubated with stoichiometric amounts of bDV RNA (2 μM) for 45 minutes, centrifuged and total protein and the resuspended pellets (in Buffer A) were loaded on a 12% SDS-PAGE. Lane 1, total lysozyme (2 μM); lane 2, reduced denatured lysozyme + bDV RNA (pellet); lane 3, lysozyme + 20 mM DTT + bDV RNA (pellet). (**C**) Filter binding studies performed to determine the time course of interactions of ^32^P bDV RNA with 2 μM lysozyme at two different experimental conditions: The percentage of ^32^P count retained on the filter with respect to the total count incorporated in the RNA (materials and methods) is plotted as a function of time when either lysozyme in presence of 20 mM DTT 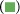 or reduced-denatured lysozyme 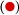 was incubated with ^32^P bDV RNA. The molar ratio of protein: bDV RNA was 1:1.

The observed effect of bDV RNA on lysozyme aggregation (stated above) however appeared contradictory to our earlier observation in which lysozyme aggregation during its refolding from the reduced denatured state was suppressed in presence of the ribosome or the *in vitro* transcribed bDV RNA^19^. Preliminary studies have been performed to address this contradiction. As shown in Fig. 6Ai and ii, a small proportion of the ribosomal proteins and RNA constitutes the residual aggregate formed even when the aggregation of reduced denatured lysozyme is suppressed in presence of the ribosome. Also in the presence of bDV RNA the lysozyme aggregates that are formed during refolding of reduced denatured lysozyme are associated with the RNA although at a much lower level than that observed with lysozyme in presence of 20 mM DTT (Fig. 6Bi and ii). These studies imply that even when there is a ribosome mediated net suppression of aggregation as reported earlier^19^, a basal level of ribosome aggregation and RNA-protein aggregate formation occurs. Filter binding experiments performed to study bDV RNA-protein interaction shows sustained interaction between the RNA and the protein occurs with both reduced-denatured lysozyme and lysozyme in presence of 20 mM DTT. The time course of RNA-protein interaction observed is however distinct (Fig. 6C). These studies suggest that the effect of ribosome-protein interaction or rRNA-protein interaction can depend on whether unfolded, partially folded or aggregating proteins interact with the ribosomal RNA and these factors are being further studied.

### Effect of ribosome and bDV RNA on BCAII aggregation

To investigate whether the formation of protein-ribosome aggregates observed with lysozyme is unique to disulfide containing proteins carrying a net positive charge or is a general phenomenon, the following experiments were performed with the BCAII protein. Bovine carbonic anhydrase II (BCAII) is a small globular protein having no disulfide linkages. Since its pI is 6.93, it carries a negative charge (−2.3) at physiological pH. BCAII has been used as a model in protein folding studies and is also capable of amyloid formation^30^. The refolding yield of BCAII, when folded from its completely unfolded state, is low due to the formation of aggregation prone molten globule intermediate formed during its refolding^31^,^32^. In addition, the human homolog of the protein is associated with the disease marble brain syndrome that is manifested in carriers of point mutations in the HCAII gene^33^.

**Figure 7:**
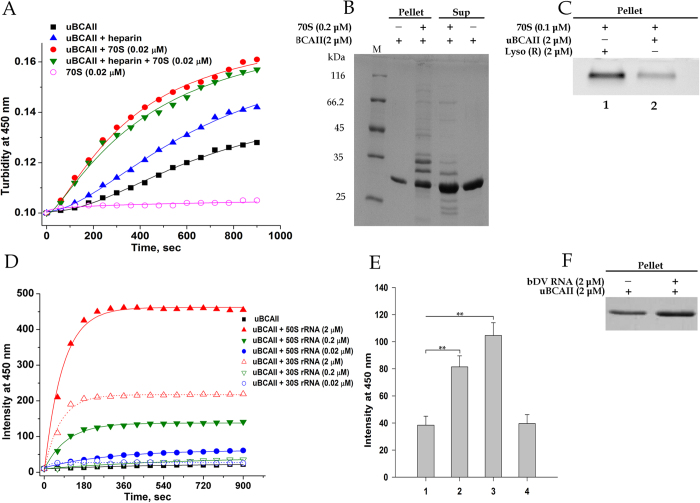
Effect of ribosome and bDV RNA on BCAII aggregation. Aggregation of uBCAII was monitored by measuring time course change in turbidity in presence of rRNA and 70S. The aggregates were also analysed using SDS-PAGE and agarose gel electrophoresis. For gel electrophoresis experiments, the samples loaded in each lane are depicted in panels above the gels. (**A**) Time course of change in turbidity of unfolded BCAII (uBCAII) in Buffer A (2 μM) in absence or presence of 70S ribosome (0.02 μM) or 1 μM heparin: only uBCAII (◾), uBCAII + heparin 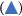, uBCAII + 70S ribosome 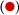, uBCAII + 70S ribosome + heparin 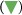, 70S + 0.06 M guanidine hydrochloride 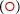. (**B**) Unfolded BCAII (uBCAII) (2 μM) was incubated in the presence or absence of 0.02 μM 70S ribosome for 20 minutes and centrifuged. The resuspended pellet in Buffer A and the supernatant (40 μl each) were loaded on a 12% SDS-PAGE. (**C**) 1% agarose gel shows the amount of rRNA in the aggregate when 70S (0.1 μM) was incubated in presence of lysozyme (2 μM) under reducing condition or uBCAII (2 μM) under refolding conditions for 60 minutes. (**D**) Time course change in scattering intensity of uBCAII (2 μM) in presence of stoichiometric and sub-stoichiometric concentrations of 50S rRNA and 30S rRNA. The aggregation process of uBCAII (◾); uBCAII + 50S rRNA [0.02 μM 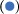, 0.2 μM 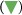, 2 μM 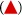]; uBCAII + 30S rRNA [0.02 μM 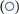, 0.2 μM 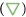, 2 μM 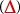] were monitored. (**E**) Bar graph representing the increase in light scattering intensity in 20 min when uBCAII (2 μM) in Buffer A was incubated in absence of any modulator (1) or in the presence of equimolar concentration of bDV RNA (2), RNA1 (3) and RNA2 (4) sub domain. Statistical significance is shown by **(p < 0.001, one-way ANOVA, N = 5) compared to control (1). (**F**) Unfolded BCAII (2 μM) was incubated in the absence or presence of bDV RNA (2 μM), centrifuged and the resuspended pellet was loaded on a 12% SDS-PAGE.

When BCAII is denatured with 6 M GuHCl and diluted 100-fold such that the final protein concentration is 2 μM, the protein undergoes aggregation as revealed by both the increase in turbidity and light scattering at 450 nm (Fig. 7A and D). As with lysozyme, a significant increase in turbidity at 450 nm was observed when unfolded BCAII (uBCAII, 2 μM) under refolding conditions (methods section) was incubated in presence of 0.02 μM ribosome and this increase was refractory to the presence of the polyanion heparin (Fig. 7A). SDS-PAGE analysis showed the presence of ribosomal components in the aggregate (Fig. 7B). To compare of the extent of ribosome aggregation induced by lysozyme under reducing conditions and uBCAII under refolding conditions, agarose gel electrophoresis was performed to detect rRNA in the aggregate. As shown in Fig. 7C, when lysozyme and uBCAII, both at concentration 2 μM, under reducing and refolding conditions respectively were incubated with 0.1 μM ribosome, the rRNA in the aggregate is ~3 fold higher in presence of lysozyme as compared to that of BCAII. Studies on the effect of ribosomal RNA extracted from the large and small ribosomal subunit on BCAII aggregation (Fig. 7D) showed that the large subunit rRNA was significantly more effective in stimulating BCAII aggregation compared to that of small subunit derived rRNA at all the stoichiometric concentrations studied. As in the case of lysozyme (stated above) a stimulated increase in light scattering was also observed when uBCAII (2 μM) was incubated with stoichiometric concentration of bDV RNA, RNA1 sub-domain but not in presence of the RNA2 sub-domain (Fig. 7E). It is to be noted that the two RNA sub-domains of bDV RNA of comparable sizes (240 nucleotides in RNA1 and 385 nucleotides in RNA2) showed differential ability in stimulating both lysozyme and uBCAII aggregation. Similar experiments performed in presence of rmf mRNA (165 nucleotides; Materials and methods) did not show significant effect on uBCAII aggregation (Supplementary Figure S2D) SDS-PAGE analysis of the insoluble precipitate confirmed that bDV RNA stimulated aggregation of uBCAII (Fig. 7F).

## Discussion

Protein misfolding and aggregation have an adverse effect on all living organisms and is also associated with several neurodegenerative diseases^34^. The protein aggregates are often implicitly classified as highly structured amyloids or amorphous aggregates. Several studies have elucidated the molecular basis underlying the cytotoxicity of protein aggregates. Although the fibrillar amyloid aggregates are believed to be toxic, recent evidences indicate at the toxicity of non-fibrillar aggregates. The granular non-fibrillar aggregates of Aβ (1–40) have been identified as possible toxic species in Alzheimer’s disease^35^. A recent study has also shown that the fibrillar aggregates of hen egg white lysozyme, formed under alkaline conditions, could be converted to amorphous aggregates in the presence of Cu(II) and these aggregates are more toxic compared to the fibrils alone^36^. Our present studies on the effect of aggregation of lysozyme and BCAII on empty non-translating ribosomes demonstrate that the partial unfolding or aggregation of these proteins is capable of inducing aggregation of ribosomal components.

As stated earlier lysozyme forms amorphous aggregates under reducing conditions used in our study^23^. The ‘misfolded’ nature of such partially folded and aggregated proteins might lead to exposure of hydrophobic residues and a vast array of amino acid side chains, some of which may also mimic native protein surfaces. These misfolded species might be able to interact inappropriately with binding partners like the membranes and other cellular components^37^. Hence, the nonspecific interaction of partially folded or aggregated lysozyme with the large number of ribosomal proteins that constitute the ribosome would seem to be imperative. Our studies however demonstrate that electrostatic interaction between the protein and the negatively charged rRNA component of the ribosome plays a significant role in inducing aggregation of ribosomal components by the protein aggregates. The role of electrostatics in the interaction between biological macromolecules is well documented. In addition to protein aggregation, the electrostatic aggregation of biological polyelectrolytes due to macroion-polyanion interaction has been shown to be important in a number of disease states. For example in Parkinson’s disease histones promote the aggregation and fibrillation of α-synuclein^38^ and in cystic fibrosis anionic polymers like DNA and F-actin bind to and sequester antibacterial proteins like lysozyme^39^,^40^. In studies of lysozyme-actin coaggregation, native lysozyme was involved in the aggregation process^41^. The ribosome –lysozyme aggregation process observed here however requires either partial unfolding or aggregation of lysozyme (Figs 1C and 3C). Our studies also imply a contribution of a non-electrostatic specific interaction between the protein and the ribosome in the lysozyme induced ribosome aggregation. Under the same conditions in which ribosome-protein aggregation is observed, the ribosomal RNA and *in vitro* transcribed RNA corresponding to domain V of 23S rRNA (bDV RNA) stimulate lysozyme aggregation. The aggregation of the non-disulfide containing protein BCAII also induces ribosome aggregation indicating that the induction of protein aggregation is a general phenomenon and not specific for disulphide containing proteins. The large subunit rRNA could also stimulate BCAII aggregation. In this context reference can be made of earlier studies demonstrating that the total RNA extracted from yeast or bovine liver can promote the assembly of the Tau protein into the Alzheimer’s disease associated paired helical filaments (PHFs). The cellular RNA isolated from neuroblastoma cells (N2a RNA) is also capable of inducing accumulation of Prp-RNA aggregates both *in vitro* and *in vivo*^42^,^43^.

As stated earlier, the ability of the bacterial ribosome to significantly improve the refolding yield of proteins is well established. It is to be noted that in these studies the ribosome and the substrate proteins were present in stoichiometric concentrations under conditions conducive to their folding. In our studies the effect of proteins undergoing aggregation on non-translating empty ribosomes has been analysed. This situation mimics the *in vivo* stress conditions in which there is an increase in population of both non-translating ribosomes (due to translational suppression) and protein aggregates^44^,^45^. Our studies also demonstrate that even when there is a ribosome mediated net suppression of aggregation, as reported earlier^19^, a basal level of ribosome aggregation and RNA-protein aggregate formation occurs. It is possible that although the domain V of 23S rRNA might be involved these varied aspects of the protein-RNA and hence protein-ribosome interaction, the nature and specificity of the interactions involved and the environment in which they occur might be distinct. In addition the diversity of cellular mechanisms that can be influenced by PFAR is evident from a recent study in which a direct link between PFAR and prion propagation has been demonstrated *in vivo*^18^. The outcome of ribosome protein interaction can depend on whether unfolded, partially folded or aggregating proteins interact with the ribosomal RNA^6^,^19^ and these factors need to be further studied. The role of the ribosome associated chaperones in preventing protein induced ribosome aggregation also needs to be analysed.

The *in vivo* accumulation of protein aggregates that are predominantly composed of ribosomal components has been reported earlier. Mutant yeast cells lacking gene for Tsa1 protein (a ribosome associated molecular chaperone having peroxidase activity) were found to accumulate aggregated proteins and this was exacerbated when the cells were subjected to reductive stress in presence of 20 mM DTT^46^. The analysis of the protein aggregates revealed that they are predominantly composed of ribosomal proteins^47^,^48^. In addition, deletion of ribosome associated chaperone network (Hsp70/Hsp40 system SSB-RAC and the nascent chain polypeptide associated complex) in yeast cells cause conditional loss of cell viability under stress conditions. The cells lacking NAC and SSB show a synergistic defect in the folding of newly synthesized proteins and their absence causes significant aggregation of a variety of nascent polypeptides. A strong reduction in 80S ribosome and polysome peaks together with aggregation of ribosomal biogenesis factors and ribosomal proteins is observed in these cells^49^. In another set of studies, neuronal death that occurs during focal brain ischemia has been attributed to irreversible destruction of the protein synthesis machinery. Protein aggregation after brain ischemia is virtually an irreversible process^50^,^51^. Since ATP depletion following ischemia disables the chaperone and degradation machinery, the nascent polypeptides are unable to fold or degrade. This ultimately leads to irreversible aggregation of translational complex components like the ribosomes, its associated nascent polypeptides, translational initiation factors and co-translational chaperones^50^,^51^. These studies have also demonstrated that the disruption of assembly and processing of new ribosomes is responsible for the observed phenomenon, although the exact mechanism of ribosomal aggregation still remains to be identified. Our *in vitro* studies suggest that the misfolded aggregation-prone proteins, whose cellular levels are likely to increase under all the aforementioned conditions, might target the non-translating ribosomes or its subunits^47^,^48^,^49^,^50^,^51^. In addition to this, a recent study has shown that impairments in protein synthesis in which the polyribosome complex is adversely affected may be one of the earliest neurochemical alterations in Alzheimer’s disease^52^,^53^. Further, as stated earlier, reduction in translation in an *in vitro* assay due to the addition of tau protein oligomers also points towards the targeting of the translating machine during aggregate mediated cellular toxicity^25^.

In this context, a recent review by Yang *et al*.^54^ needs special mention. This review discusses several emerging evidences demonstrating that sequestration of cellular interacting partners by protein aggregates contributes to pathogenesis in neurodegenerative diseases. Of the mechanisms proposed, the RNA assisted sequestration and the sequestration of molecular chaperones by protein aggregate that results in loss of biological functions are relevant in context of our observations. As has been discussed in this review, while some sequestrations are nonspecific, some are mediated by specific interactions. The ability of the unfolded protein to interact specifically with rRNA residing in the ribosome forms the basis of PFAR. Such interactions with the partially unfolded regions of proteins might be able to create a high local concentration of the protein and hence trigger its aggregation. This process might be able to initiate the sequestration or trapping of the ribosome itself leading to aggregation of its components. In case of proteins that carry a net positive charge at physiological pH, electrostatic interaction with the negatively charged ribosome surface might also have a nonspecific but significant contribution in the sequestration process. Whether the protein-RNA interactions involved here might be distinct from that necessary for the PFAR activity and whether other ribosomal components also contribute to this process needs further characterization. Further analysis of the precise composition of the ribosomal components (rRNA and ribosomal proteins) that constitute the ribosome-protein aggregate also needs to be performed. In addition, the relative ability of disease associated amyloid aggregates and amorphous protein aggregates to induce ribosomal aggregation also needs to be studied. However, this study raises the possibility that the sequestration of the ribosome due to protein aggregate-rRNA interaction might affect the integrity of the ribosome thereby leading to cytotoxicity.

## Experimental Procedures

### Materials

Bovine carbonic anhydrase II (BCAII), hen egg white lysozyme, GuHCl, DTT, Tris-base and total *E.coli* tRNA (f-Met deacylated tRNA) were purchased from M.P Biomedicals. Heparin and the yeast *Saccharomyces cerevisiae* 80S ribosome were kind gifts from the laboratories of Dr. S. N. Kabir and Dr. Jayati Sengupta (IICB). Nitrocellulose filter was purchased from Millipore and reagents for molecular biology like T7 RNA Polymerase and RNase free DNase I were purchased from Fermentas. All the other chemicals used were local products of analytical grade. Data analysis for turbidity measurements and light scattering experiments was performed using OriginPro8 software and statistical analysis for bar diagrams performed using Sigma Plot 13. The densitometry analysis was done using Quantity One (Bio-Rad).

### Buffers

The buffers used in this study are the following: Buffer A: 50 mM Tris-HCl (pH 7.5), 10 mM MgCl_2_, 100 mM NaCl^19^,^28^; Buffer P, 50 mM Tris-HCl (pH 7.5), 7 mM MgCl_2_, 30 mM KCl, 70 mM NH_4_Cl^28^.

### Preparation of ribosome and *in vitro* synthesis of Ribosomal RNA

The DNA corresponding to domainV of the *E. coli* large subunit RNA (bDV RNA) and bovine mitochondrial large subunit RNA (mDV RNA), cloned in plasmid pTZ57R/T were kind gifts from the laboratory of Professor Chanchal Dasgupta (University of Calcutta). The mDV RNA has a truncated RNA2 sub-domain and therefore shows a delay in releasing the bound protein^10^. The DNA corresponding to RNA1 portions of *E. coli* domainV was cloned into the pTZ57R/T vector downstream to the T7 Polymerase promoter. The RNA corresponding to mDV RNA, bDV RNA, RNA1 and RNA2 were synthesized by run-off transcription and prepared as described earlier^10^,^19^,^28^,^55^. Experiments were also performed on the effect of a 165 bp mRNA (obtained by *in vitro* transcription of the *E.coli* Ribosome modulation factor gene cloned in pET 28a vector available in the laboratory) on lysozyme and BCAII aggregation.

Ribosomes were purified from *E. coli* MRE 600 cells and the ribosomal subunits were prepared as described in ref. 8. The ribosomal RNA (rRNA) from 50S and 30S subunits was extracted by lithium chloride as described in ref. 56.

### Binding of free70S with total *E. coli* tRNA

The binding of total *E. coli* tRNA to the free or empty ribosome was performed in Buffer-P as described earlier^28^. This tRNA bound 70S ribosome was then used in lysozyme aggregation studies.

#### Light scattering measurements

Partially unfolded lysozyme aggregates under reducing condition and is a widely used model for protein aggregation as stated earlier. In our studies the aggregation behaviour of lysozyme and bovine carbonic anhydrase II was monitored in presence of the ribosome and bDV RNA by turbidity measurements at 450 nm in Hitachi spectrophotometer (U-1900) and by scattering measurements in a Hitachi spectrofluoremeter (F-2700) with excitation and emission set at 450 nm respectively in Buffer A. Since the ribosome shows light scattering at 450 nm, all the time course studies in presence of the ribosome were done by measuring the change in turbidity at the mentioned wavelength.

### Studies with the ribosome and ribosomal RNA

Hen egg white lysozyme (2 μM or 10 μM or 65 μM) was incubated in 20 mM DTT (for all experiments performed under reducing conditions) in the absence or presence of 70S ribosome (1 μM or 0.1 μM) and rRNA (0.02 μM–2 μM). For studies with BCAII the protein (200 μM) was denatured with 6 M guanidine hydrochloride and 3.5 mM EDTA for 3 hours and then diluted 100 fold in the absence or in the presence of ribosome (0.02 μM except mentioned in the figure legend) and rRNA (0.02 μM–2 μM) in Buffer A to study the aggregation process. The denatured and reduced lysozyme was prepared by incubating lysozyme (200 μM) with 6 M guanidine hydrochloride and 100 mM DTT for 3 hours. The aggregation process of denatured and reduced lysozyme was studied by 100 fold dilution of the denaturants in the absence or in the presence of ribosome (0.2 μM). Protein aggregation was also monitored in the presence of rRNA extracted from the ribosomal subunits using lithium chloride. The rRNAs were present different stoichiometric ratios with respect to the protein as indicated in the Figures.

### Studies with bDV RNA

To study the effect of bDV RNA on 2 μM of lysozyme (under reducing conditions) and or 2 μM denatured BCAII (under refolding conditions) were incubated with *in vitro* transcribed bDV RNA (at concentrations indicated in the Figure) or stoichiometric concentrations of RNA1, RNA2 and mDV RNA and the aggregation was followed using light scattering measurements as stated above. Similar experiments were also performed to study the effect of equimolar concentrations of heparin (2 μM) on lysozyme aggregation. The increase in light scattering was followed for a period of 45 min and 20 min for reduced lysozyme or denatured BCAII respectively and the increase in scattering intensity in presence of heparin and the different RNA variants were plotted as bar diagrams for comparison. The light scattering experiments performed with the rRNA and the bDV RNA variants were repeated five times and the data represents the average of all these experiments.

#### Gel electrophoretic analysis of the aggregate

The proteins (lysozyme and BCAII) were incubated with the 70S ribosome, 80S ribosome, bDV RNA for 45 minutes or as mentioned in the figure legend (lysozyme) or for 20 minutes (BCAII). The insoluble aggregates formed were separated by centrifugation at 21,380 g for 20 minutes at 4 °C. The pellet was resuspended in the Buffer A. The supernatant was concentrated using Amicon Ultra-10K filter. The resuspended pellet and supernatant were loaded on 12% SDS –PAGE for analysis of the proteins constituting the aggregates. The RNA incorporated in the aggregate was analysed by 1% agarose gel electrophoresis of the pellet resuspended in Buffer A containing 1 M urea. For all agarose gel experiments, the concentration of 70S or 80S ribosome used was 0.1 μM.

For the control experiment where the effect of native BCAII was tested on lysozyme aggregation, 10 μM lysozyme in presence of DTT was incubated with or without 2 μM, 1 μM, 0.5 μM BCAII in Buffer A containing 20 mM DTT for 45 minutes at room temperature and centrifuged at 21,380 g for 20 minutes. The pellet fractions were resuspended in 40 μl of Buffer A and the supernatant fractions were concentrated as described above before loading on a 12% SDS - PAGE.

#### Studies on protein-RNA interactions

Filter binding studies were performed as described in refs 4 and 52. The ^32^P-labelled bDV RNA was obtained by *in vitro* transcription (stated above) in presence of a ^32^P-UTP followed by DNase I digestion and precipitation using ethanol. Lysozyme was incubated with equimolar ^32^P-bDV RNA in the presence of 20 mM DTT for various time intervals and filtered through pre-soaked nitrocellulose filter paper (Millipore) with pore size of 0.22 μm. The filters were dried and ^32^P counts were taken in a liquid scintillation counter (Perkin Elmer). The RNA bound to the protein was retained on the filter while the free RNA passed through it. The percentage of radioactivity retained on the filter paper, calculated considering the radioactive count incorporated in the total RNA to be 100%, was plotted against time.

#### Electron Microscopy

Lysozyme (10 μM) was incubated with 70S ribosome (0.1 μM) for different time intervals (1 min, 5 min, 10 min, 30 min and 45 min from the initiation of incubation) in presence of 20 mM DTT and the samples were diluted 10-fold in Buffer A before preparation of EM grids were prepared immediately as stated in ref. 19. Imaging of aggregation in the refolding samples was done by using a transmission electron microscope (FEI Tecnai12BioTwin). Control experiments were performed in which, 70S ribosome and lysozyme were incubated separately with 20 mM DTT for 1 hour and observed under identical conditions.

## Additional Information

**How to cite this article:** Pathak, B. K. *et al*. Sequestration of Ribosome during Protein Aggregate Formation: Contribution of ribosomal RNA. *Sci. Rep.* **7**, 42017; doi: 10.1038/srep42017 (2017).

**Publisher's note:** Springer Nature remains neutral with regard to jurisdictional claims in published maps and institutional affiliations.

## Supplementary Material

Supplementary Information
