# Supplementary material for: Sequestration of Ribosome during Protein Aggregate Formation: Contribution of ribosomal RNA
Source: Sci Rep. 2017 Feb 7;7:42017. doi: 10.1038/srep42017 (PMC5294636; doi:10.1038/srep42017)
Supplement: Supplementary Information [file srep42017-s1.pdf]

Title

Sequestration of Ribosome during Protein Aggregate Formation: Contribution of ribosomal RNA

Bani K. Pathak[1], Surojit Mondal[1], Senjuti Banerjee[1], Amar Nath Ghosh[2] and Chandana Barat[1*]

[1] Department of Biotechnology, St. Xavier's College, Park Street, Kolkata-700016, West Bengal, India.

[2] National Institute of Cholera and Enteric Diseases P-33, C.I.T. Road, Scheme XM, Beleghata, India

* Corresponding author. Email: [chandanasgb@yahoo.com]

Supplementary Figure S1

In the control experiment where the effect of native BCAII was tested on lysozyme aggregation, 10 μM lysozyme in presence of DTT was incubated with or without 2 μM, 1 μM, 0.5 μM BCAII in Buffer A containing 20 mM DTT for 45 minutes at room temperature and centrifuged at 21,380g for 20 minutes. The pellet fractions were resuspended in 40 μl of Buffer A and the supernatant fractions were concentrated as described above before loading on a 12% SDS - PAGE.

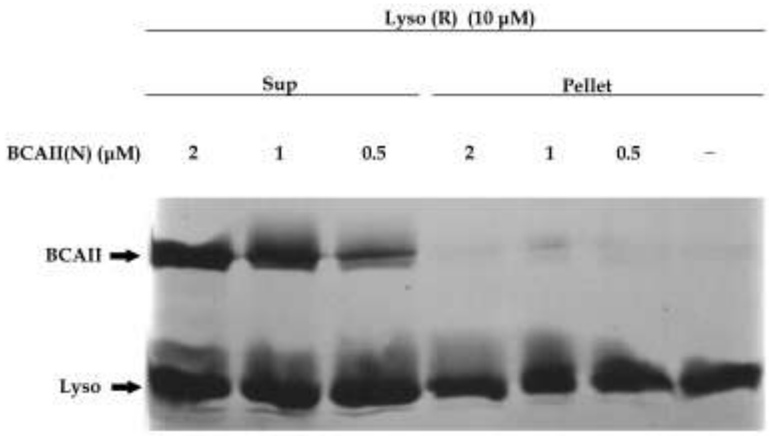

Supplementary Figure S1 legend: Effect of native BCAII on lysozyme aggregation

10 μM of lysozyme was incubated with and without 2 μM, 1 μM and 0.5 μM of native BCAII in presence of 20 mM DTT for 45 minutes at room temperature in Buffer A and centrifuged. The concentrated supernatants and the resuspended pellets in Buffer A were analysed on 12 % SDS PAGE. The lanes from left to right contain: Lysozyme + DTT + 2μM of BCAII (supernatant), lysozyme + DTT + 1μM of BCAII (supernatant), lysozyme

+ DTT + 0.5 μM of BCAII (supernatant), lysozyme + DTT + 2μM of BCAII (pellet), lysozyme + DTT + 1μM of BCAII (pellet), lysozyme + DTT + 0.5 μM BCAII (pellet), lysozyme + DTT (pellet).

Supplementary Figure S2

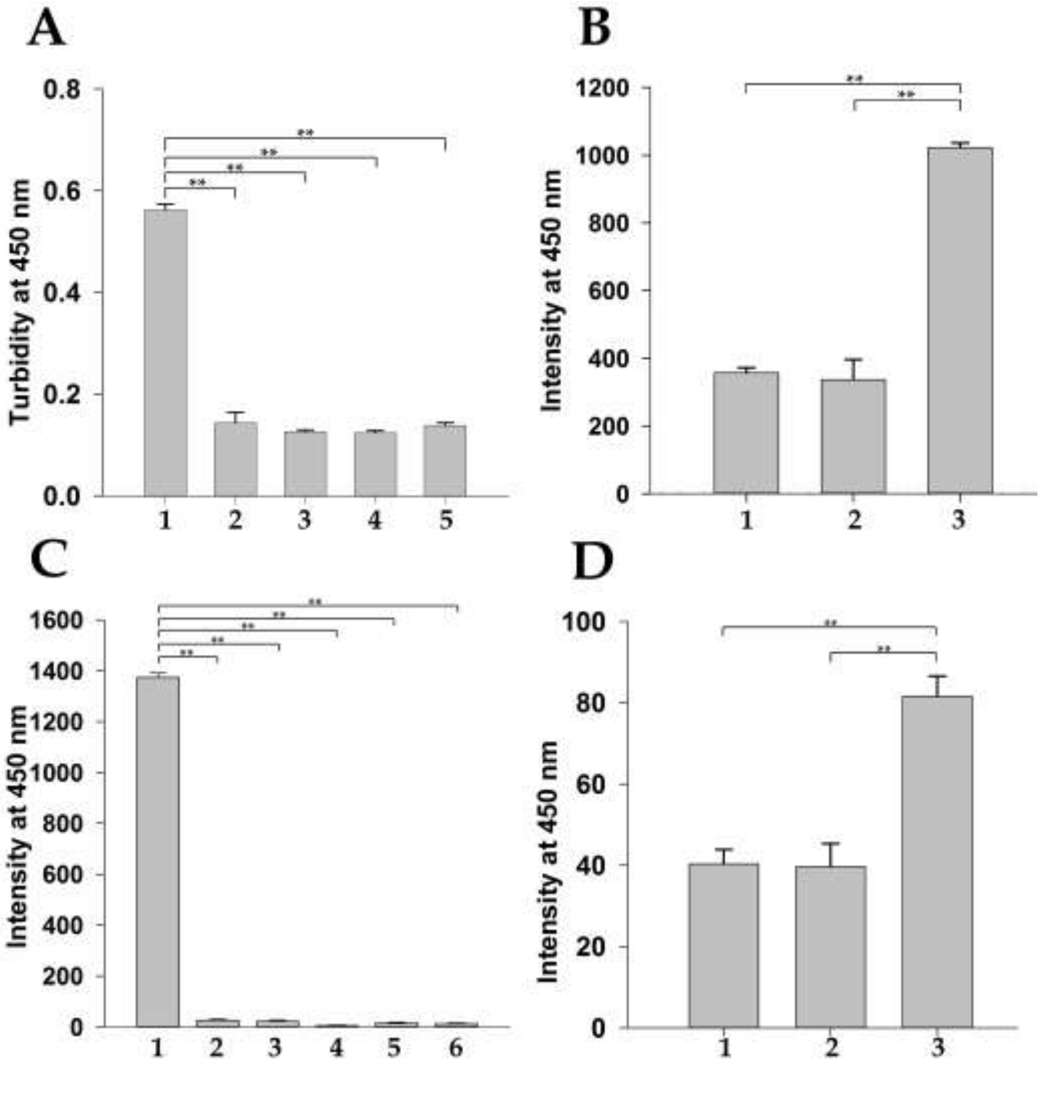

Supplementary Figure S2 legend: Control experiments with Lysozyme and BCAII

A) Increase in turbidity in 45 min when 10 µM Lyso (R) (Lysozyme + 20 mM DTT) was incubated in the presence of 0.1µM of 70S, 0.2 µM of mRNA (45 nucleotides) or10 µM of tRNA. (1) Lyso(R) + 70S, (2) Lyso(R) + 70S + tRNA, (3) Lyso(R) + mRNA, (4) Lyso(R) + tRNA,(5) Lyso(R) + mRNA + tRNA. Statistical significance is shown by ** ($p < 0.001$, one-way ANOVA, N = 3).

B) Increase in light scattering intensity in 45 min when 2µM Lysozyme in 20 mM DTT is incubated in presence of 2 µM mRNA (165 nucleotides) and 2 µM bDV RNA. (1) Lyso(R), (2) Lyso(R)+mRNA, (3) Lyso(R) + bDVRNA. Statistical significance is shown by ** ($p < 0.001$, one-way ANOVA, N = 3).

C) Increase in light scattering intensity in 45 min when 2 µM rRNA, 2 µM bDV RNA or mDV RNA is incubated in presence of 20 mM DTT (2) 50S rRNA, (3) 30S rRNA, (4) bDV RNA, (5) mDV RNA, (6) RNA1. Increase in light scattering when 2µM Lysozyme reduced in 20 mM DTT is incubated in presence of 50S rRNA (1) is also shown here. Data are presented as means ± SEM; ** P< 0.001in one –way ANOVA (N=3).

D) Increase in light scattering intensity in 20 min when 2µM uBCAII is incubated in presence of 2 µM mRNA (165 nucleotides) and 2 µM bDV RNA. (1) uBCAII, (2) uBCAII + mRNA, (3) uBCAII + bDV RNA. Statistical significance is shown by ** ($p < 0.001$, one-way ANOVA, N = 3).
